# The Low Velocity Impact Response of Shape Memory Alloy Hybrid Polymer Composites

**DOI:** 10.3390/polym10091026

**Published:** 2018-09-14

**Authors:** Hao Li, Jingbiao Liu, Zhenqing Wang, Zhengwei Yu, Yanfei Liu, Min Sun

**Affiliations:** 1 College of Aerospace and Civil Engineering, Harbin Engineering University, Harbin 150001, China; lihao0202@hrbeu.edu.cn (H.L.); liujingbiao@hrbeu.edu.cn (J.L.); yzw0514@hrbeu.edu.cn (Z.Y.); sunmin@hrbeu.edu.cn (M.S.); 2 State Key Laboratory of Tribology, Tsinghua University, Beijing 100084, China; yanfeiliu@hrbeu.edu.cn

**Keywords:** polymer composites, shape memory alloy, low velocity impact

## Abstract

Polymer composites are sensitive to impact loading due to their low impact resistance. Shape memory alloy (SMA) wires have been used to improve the impact resistance of the polymer composite materials because of their unique superelasticity performance. In this study, a new SMA hybrid basalt fiber-reinforced polymer composite embedded with two perpendicular layers of superelastic SMA wires is designed and the low-velocity impact behavior is experimental investigated. For contrast, the conventional polymer composite without SMA wires is also tested as the reference laminate. The tests are carried out at three different impact energy levels (30, 60 and 90 J). Moreover, to find out indications for manufacturing of SMA hybrid composites with high impact resistance, four different SMA wires embedded modes are investigated. Visual inspection and scanning electron microscope methods are adopted to identify the damage modes of the impacted samples. Results show that the impact resistance of the hybrid laminates is improved due to the hybridization of SMA wires. The most effective impact resistance of the SMA hybrid composites can be obtained by incorporating the SMA wires with one layer between the front two plies and another layer between the bottom two plies into the composite structure.

## 1. Introduction

Due to the superior properties of high stiffness, strength and very low weight, polymer composites have been widely used as structural components in engineering fields such as aerospace and automotive industry in recent years. Composites are unavoidably subjected to impact loads during their lifetime, for the poor through thickness performance, composite laminates are sensitive to the impact loading. The existence of internal damage, especially the occurring of barely visible delamination [1,2,3,4,5], reduces the integrity of the entire structure severely and thereby degrades the mechanical properties of the laminates disastrously. Therefore, the improvement of composite resistance to impact has been an important issue for the better use of composite laminates. In recent decades, lots of research have been done on the enhancement of composites impact resistance. The solutions used mainly contain matrix amelioration [6,7], interlayer toughening [8,9,10], Z-direction (through the thickness) reinforcement [11,12] and hybrids [13,14]. With these treatments, the matrix toughening is reinforced, the weakness of interface is reduced, the stress transmission in composites is more effective and the stiffness of plates is improved, thus providing the composite laminates with higher impact resistance. One alternative method is to incorporate the superelastic shape memory alloy (SMA) fibers into the composite laminates. Superelastic SMA holds nearly high constant stress lever over large recoverable strain and has unusual high failure strain, which makes it more suitable to reinforce the composites performance than other metals [15,16].

The improvement of impact resistance is primarily due to the superelastic property of SMA wires. The superelasticity is characterized by the stress induced martensitic phase transformation presenting a plateau region in the stress-stress curve [17], which renders the SMA capability to absorb a considerable amount of energy, thereby reducing the impact damage in the composites embedded with SMA wires. Extensive research has been made on low velocity impact response of SMA hybrid composites [18,19,20,21,22]. Paine and Rogers [18] have studied the low velocity impact response of brittle graphite/bismaleimide composites embedded with a small number of superelastic SMA wires. The results show that the impact resistance of the hybrid composites is improved as the hybrid composites are not perforated compared to the reference composites. The delamination area has a decrease of 25 percent due to the addition of SMA wires. Carbon fiber reinforced poly (butylene terephthalate) composites incorporated with SMA wires subjected to low-velocity impact have been investigated by Aurrekoetxea et al. [19]. The work indicates that SMA fibers do benefit the impact tolerance as the maximum allowable load increases. It is pointed out that the large energy absorbing capability and high reversible force of superelastic SMA contribute to the higher impact resistance of hybrid composites. Kang and Kim [21] have made a research on the impact response and post-impact residual properties of glass fiber composites embedded with SMA under low temperature. The research results show that the SMA laminates are more affected by the temperature than the base laminates and also present higher impact tolerance. Moreover, the impact damage has a great influence on the residual strength regardless of temperature, while the flexural modulus is less affected by the existence of damage. In the work of Khalili et al. [22], a complete model is applied to investigate the influence of pre-stress SMA wires on the smart hybrid composite plates subjected to low velocity impact. The work demonstrates that the global impact behavior of the unidirectional hybrid composite plates is improved by the addition of SMA wires. Furthermore, they find that the volume fraction of SMA wires has a considerable effect on the maximum contact force time, maximum contact force and contact time. In contrast to the no positive influence on the thick laminates, embedding SMA wires into the thin walled structures makes a beneficial influence on the impact tolerance of the traditional composites. These efforts mentioned above mainly focus on the impact behavior of glass or carbon fiber reinforced composites embedded with SMA wires, there is less research on the SMA hybrid basalt fiber-reinforced composite laminates. Moreover, the SMA hybrid composites laminates studied mostly include only one layer of SMA wires. Therefore, more work is needed to investigate the influence of the embedded SMA wires on the impact response of the basalt fiber-reinforced polymer composites.

In this paper, to enhance the impact resistance of conventional basalt fiber-reinforced polymer composites, a new SMA hybrid polymer composite embedded with two perpendicular layers of superelastic SMA wires is designed. To acquire hybrid laminates with high impact tolerance, four different embedded modes are studied. The low-velocity impact behavior of composites with and without SMA wires is evaluated by analyzing the contact force-time, displacement-time and energy-time curves. Moreover, three different incident energy levels (30, 60 and 90 J) are studied. Destructive and nondestructive methods are adopted to identify the damage modes. Based on the impact results, indications can be found out for manufacturing of SMA/basalt hybrid polymer composites with enhanced impact performance.

## 2. Materials and Methods

### 2.1. Materials

In this work, the Vinyl ester resin is used as the matrix for the basalt fiber-reinforced polymer composites. This resin can be cured at ambient temperature with addition of accelerating agent and hardening agent. The accelerating agent used is Dimethylaniline and the Methyl Ethyl Ketone Peroxide (MEKP) is used as hardening agent. All the aforementioned materials are purchased from Ashland Inc., Lexington, KY, USA. The resin, hardening agent and accelerating agent are mixed with the weight ratio of 100:1:0.15. The Unidirectional basalt fiber cloth with single layer thickness of 0.2 mm and surface density of 280 g/m^2^ is provided by Tongxiang Mentai Reinforced Composite Material Company, Tongxiang, China. The mechanical parameters of the Vinyl ester resin and the basalt fiber cloth are represented in Table 1.

**Figure 1:**
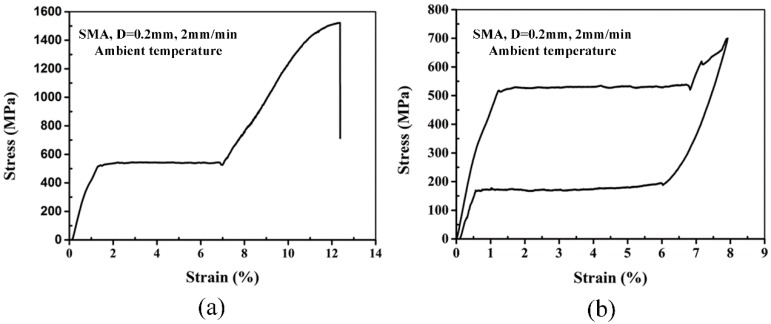
Tensile stress-strain curves of superelastic SMA wires ((**a**) Representative stress/strain curve of the tensile tests, (**b**) superelastic deformation of the SMA wire).

The superelastic 55.8 wt % Ni balance Ti wires used in this work are a commercially available NiTi alloy, ref. D0.2 (production code), purchased from PeierTech, National High-tech Zone, Jiangyin, Jiangsu, China. The SMA wires (0.2 mm diameter) have transformation temperatures, *M*_s_ = −17.6 °C, *M*_f_ = −38.2 °C, *A*_s_ = −5.7 °C, *A*_f_ = 11.2 °C. The results of tensile test on superelastic SMA wires are reported in Figure 1. Based on the stress-strain curves, the main mechanical characteristics of SMA wires are summarized in Table 2.

### 2.2. Specimen Manufacturing

**Figure 2:**
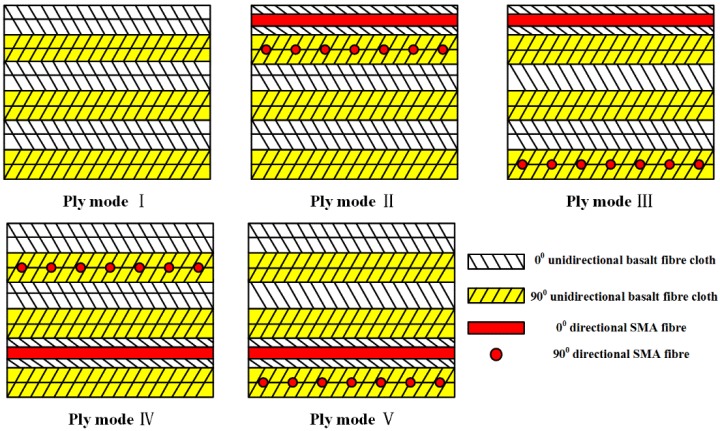
The sketches of composites with different ply modes.

In this study, unidirectional basalt fiber-reinforced composite laminates with and without SMA wires are manufactured by vacuum-assisted resin injection (VARI) process [23]. The basalt fiber-reinforced laminates without SMA are fabricated with a cross-ply configuration of [0°/0°/90°/90°]_3_, where the 0° and 90° represent the fiber orientation of each ply and the subscript 3 indicates that this lay-up is repeated three times. Lei et al. [24] have pointed out that the weak interface between SMA wires and matrix can result in a decrease of the composite performance, so treatments on the SMA surface are needed to improve the adhesion performance of SMA/matrix interface. To inhibit the debonding of SMA/matrix, efforts to enhance the interface adhesion properties have been reported [25,26,27,28,29]. In this paper, SMA wires are cleaned with acetone to remove macroscopic impurities on the alloy surface, followed by mechanical grinding and polishing with 400 grit papers to change the alloy surface roughness. After the treatments, the SMA fibers are incorporated into the composite laminates with four different modes, the sketches of ply modes are plotted in Figure 2. The ply mode І represents the lay-up of the composite without SMA, the others display the composites embedded with SMA wires. Two perpendicular layers of SMA wires with a volume fraction of 0.82% are embedded into the composites. Each layer of SMA wires with a gap of 0.3 mm is aligned along the fiber orientation of adjacent plies, which makes SMA wires well compatible with adjacent plies upon curing [30]. The specific locations of the embedded SMA wires in composites are also indicated in Table 3.

**Figure 3:**
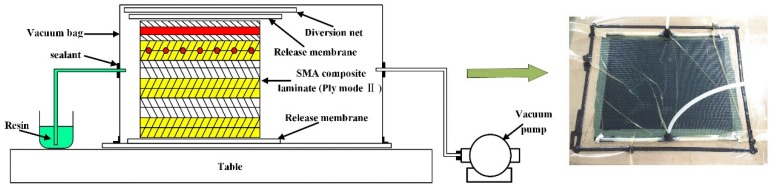
The schematic diagram of the VARI process.

The schematic diagram of the VARI method is displayed in Figure 3. During the manufacturing process, a glass plate as the holder is placed on a table. Then the hybrid composite with one layer release membrane on each side is arranged, and the diversion net is put on the top. Finally, the laminate is covered by a vacuum bag. To guarantee that the resin can flow uniformly, two delivery pipes are fixed at the entrance and exit, respectively. After the infusion of resin, the system is cured at ambient temperature and vacuum level of 600 mbar for 15 h. Specimens of 100 × 100 × 2.6 mm^3^ in dimensions are cut from manufactured plates with a diamond saw blade cutting machine.

### 2.3. Low-Velocity Impact Test

**Figure 4:**
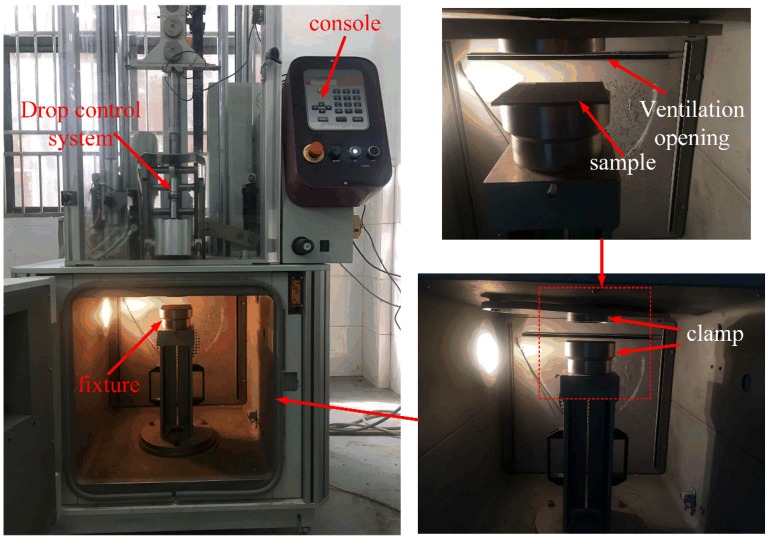
Testing setup of the low velocity impact test.

According to the ASTM D5420-2010 standard, low-velocity impact tests are conducted by Instron Dynatup CEAST 9350 (Instron, Norwood, MA, USA) drop weight impact testing machine at room temperature, as shown in Figure 4. The test system contains three parts: the pneumatic clamping fixture, a drop hammer device and a data acquisition system. Specimens are firmly fixed between two circular rings of 76 mm diameter. The steel hemispherical projectile with 16 mm diameter and 3.77 kg mass is adopted for all tests. This projectile is guided through two smooth columns, which prevents the specimens from multiple strikes. During the impact event, the impact force is measured by a load cell located just above the projectile head, and the displacement is acquired by a laser detector attached on the impact frame. In this work, three different incident energy levels, 30, 60 and 90 J are studied, which leads to the impact velocities of about 3.99 to 6.91 m/s corresponding to the common low-velocity impact cases. For each type of composites under the same impact energy, at least three specimens are tested and the mean values are determined.

### 2.4. Analysis Techniques

During the impact event, damage may occur in the impacted hybrid laminates, such as matrix cracking or crushing, fiber breaking, delamination and SMA/matrix debonding. To evaluate the type and extent of the impact damage, visual inspection and scanning electron microscopy (SEM) (Hitachi S-4300, Tokyo, Japan) methods are applied. The damage region around the impact point is selected to observe the micro-damage morphology of the impacted specimens.

## 3. Results

### 3.1. Damage Morphology of the Impacted Samples

**Figure 5:**
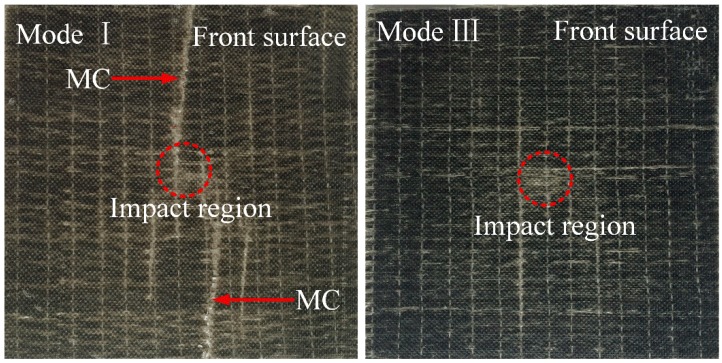
Typical damage morphology of the samples with 30 J impact. Notes: MC indicates the matrix cracking in the figure.

**Figure 6:**
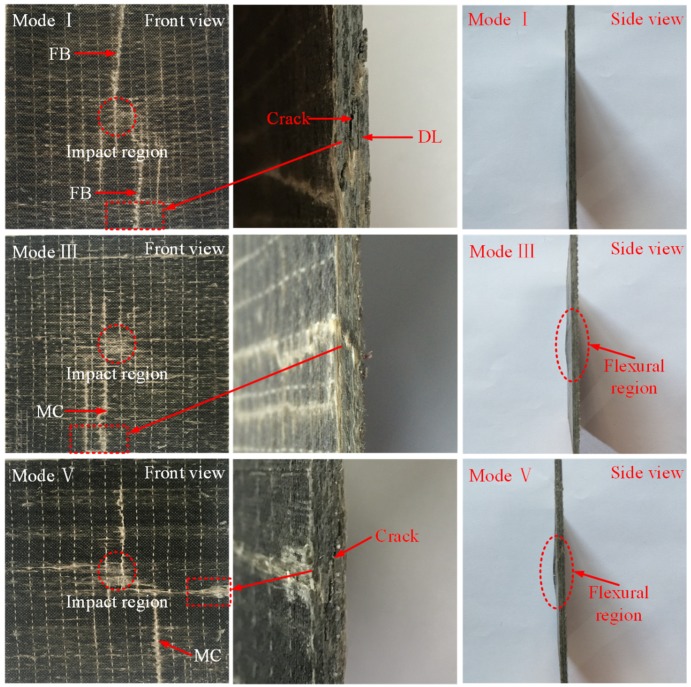
Typical damage modes of the samples with 60 J impact. Notes: in the figure, MC indicates the matrix cracking, FB represents the fiber breakage and DL illustrates the delamination.

**Figure 7:**
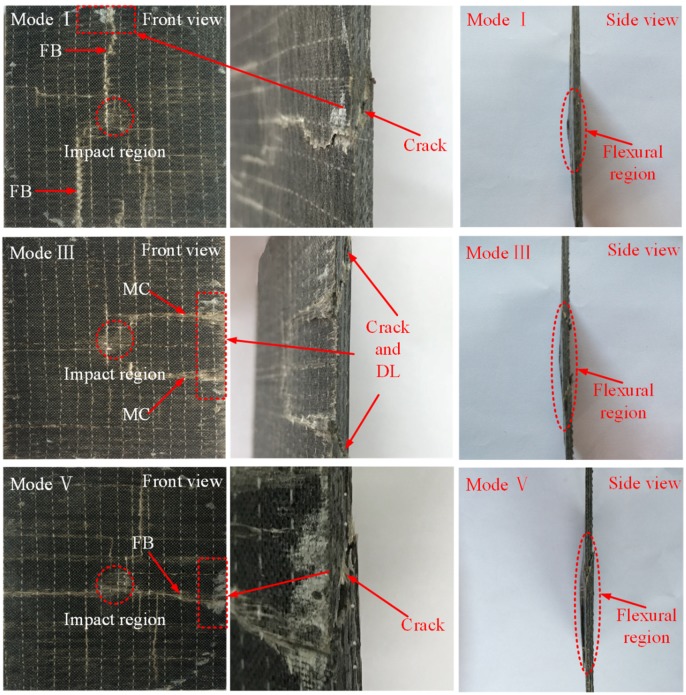
Typical damage modes of the samples with 90 J impact. Notes: in the figure, MC indicates the matrix cracking, FB represents the fiber breakage and DL describes the delamination.

The macro-damage modes of the composite laminates can be revealed by visual inspection. The typical damage morphology of the laminates impacted with 30 J is shown in Figure 5. On the contact surface, the indentation in the impact region is barely seen for both ply modes. Compared to the ply mode III laminate, it is evident that matrix cracking has occurred in the reference laminate. For 60 J impact tests, significant differences of the typical damage modes are observed in Figure 6. In the impact region, the indentation on the front surface is clearer compared with the 30 J impact events. For the laminate without SMA, fiber breakage is seen on both sides of the impact laminate. It is easily observed that a macro-crack cuts through the thickness of the laminate in the partial enlargement drawing. The delamination is also generated at the lower part of the structure. For the ply mode III laminate, matrix cracking occurs on the front surface of the impacted sample. There is no crack developing through the thickness. It is obvious that local flexural deformation appears at the center of the laminate relative to the laminate without SMA wires. For the ply mode V laminate, matrix cracking is seen on the contact surface. A crack propagates through the lower part of the structure but not reaching the front surface. A flexural region also appears in a side view of the impacted sample. Similar to the ply mode V sample, matrix cracking or fiber breakage on the front surface, and a macro-crack through the thickness are observed in the ply mode II and IV samples. Typical damage modes of the samples impacted with 90 J are reported in Figure 7. For all ply modes, the depth of indentation in the contact region is increased compared to that of the 60 J impacts. For the reference sample, two main cracks propagate more severely and almost join together at the center of the sample, resulting in catastrophic damage to the sample. A flexural region has come up in the center of the structure. For the ply mode III sample, the induced damage mainly develops at one side of the sample, macro-crack through the thickness and delamination at the upper and lower parts of the sample are observed and the flexural region has also extended. The damage induced in the ply mode II and IV samples is similar to the ply mode III sample. For the ply mode V sample, the combination of crack and delamination leads to a larger flexural region compared to that of the 60 J impacted sample.

**Figure 8:**
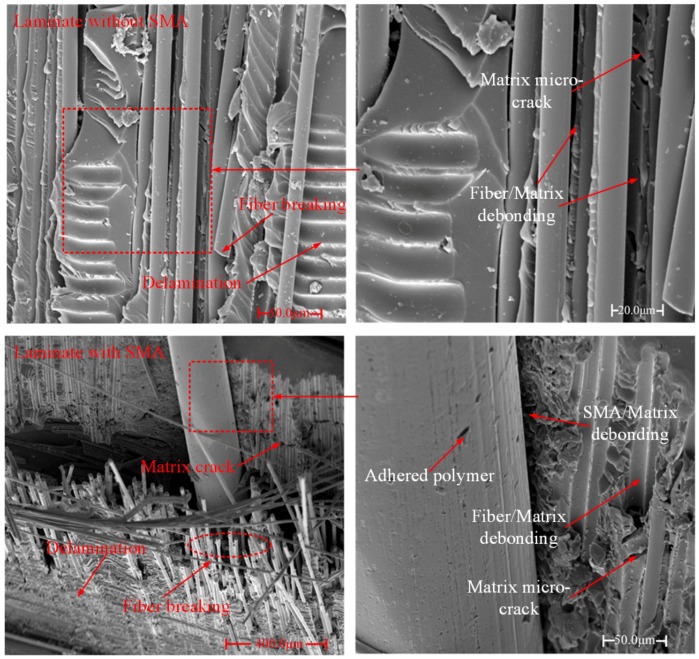
Typical damage morphology of the impacted laminates obtained by SEM.

The micro-damage morphology of the impacted samples can be obtained by SEM images, as shown in Figure 8. For the damage laminate without SMA wires, typical damage modes, including matrix micro-cracking, delamination, debonding of fibers from the polymer and fiber breakage, are clearly recorded by the SEM figures of the selected damage region. Besides the damage modes occurring in the reference laminate, the SMA-matrix debonding can be seen in the images of the impacted laminate reinforced with SMA wires. It can also be seen that a large fiber separation and breaking region is observed around the SMA wire and a crack is generated between them.

### 3.2. Impact Behavior

**Figure 9:**
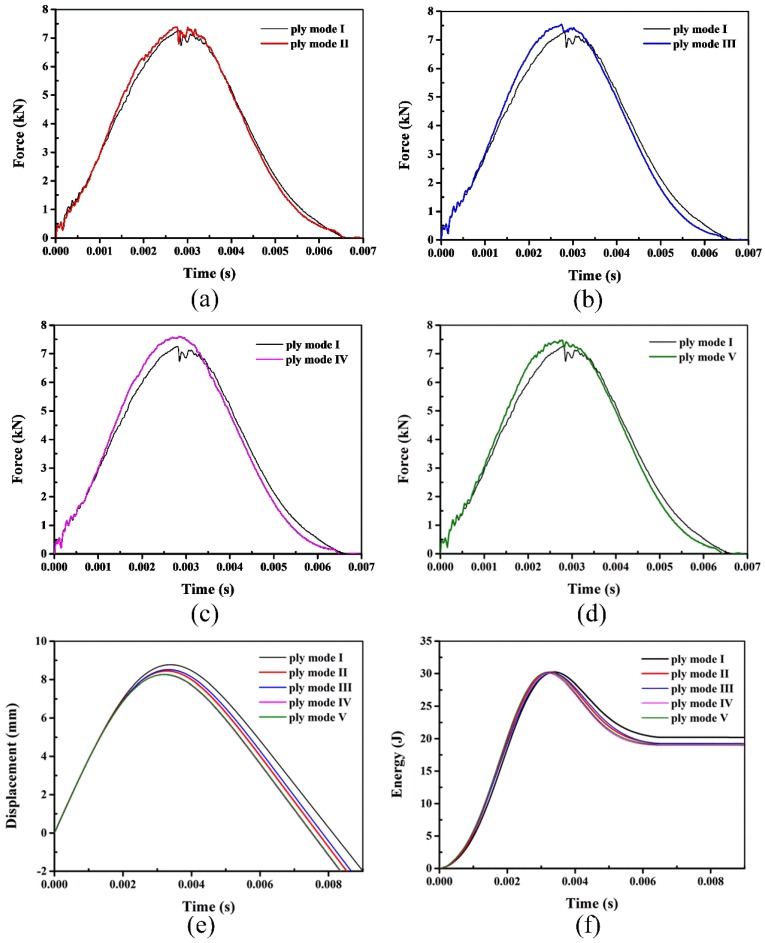
Typical impact behavior of laminates with 30 J (Figures (**a**–**d**) show the force-time curves of mode I with mode II, mode III, mode IV and mode V, respectively. Figure (**e**) and Figure (**f**) represent the displacement-time and energy-time histories of different modes, respectively).

Impact responses including contact force, displacement and absorbed energy are important to the evaluation of the impact resistance. Typical impact behavior of laminates with and without SMA wires at 30 J is shown in Figure 9. It can be seen that the shape of contact force-time curves nearly shows symmetric. For the reference laminate and the ply mode II, III hybrid laminates, there is a small load drop in the force-time curves. The matrix cracks around the contact area, as shown in Figure 5, result in the load drop in the reference laminate, while the load drop in the SMA hybrid laminates may be caused by the SMA/matrix debonding. For the mode IV and V laminates, the impact damage is insufficient to have a noticeable influence on the impact response, so there is no evident load drop in the force-time curves. The peak forces of SMA hybrid laminates are all higher than the reference laminate. Relative to the displacement-time curves, the displacement of the conventional laminate is the largest, the displacement-time history of the ply mode IV laminate is almost the same to the ply mode V and the displacement is the smallest. In the energy-time curves, the absorbed energy of the laminate without SMA is the highest. There are no significant differences of the rebound energy for the four types of hybrid laminates, which indicates that the positions of SMA wires make no great influence on the absorbed energy of hybrid laminates at 30 J.

**Figure 10:**
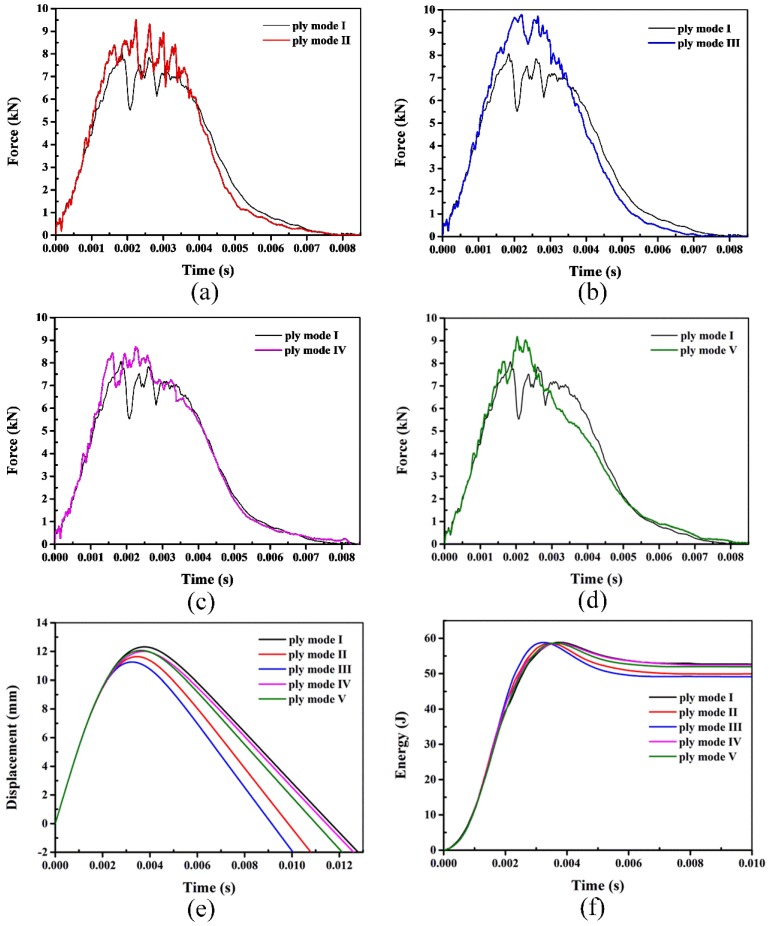
Typical impact behavior of laminates with 60 J (Figures (**a**–**d**) show the force-time curves of mode I with mode II, mode III, mode IV and mode V, respectively. Figure (**e**) and Figure (**f**) represent the displacement-time and energy-time histories of different modes, respectively).

Figure 10 shows the typical contact force-time, displacement-time and energy-time curves of laminates impacted with 60 J energy. It can be seen that laminates are not penetrated and the overall impact behavior can be divided into two phases, the pre-rebound phase and the rebound phase. During the pre-rebound phase, the force continuously increases with load increasing. For the presence of damage, such as the matrix cracking, fiber/matrix debonding and SMA/matrix debonding, the contact force rises slower. Sudden load drops occur when there is severe damage induced in the laminates, which is represented by the peaks and valleys in the force versus time history. The propagation of delamination and the formation of macro-crack through the thickness both can result in large load drops in the force-time history. In the rebound phase, the contact force continuously decreases until the separation of the impactor from the laminate. Compared to the 30 J impact, the peak forces of SMA hybrid laminates are much higher than the conventional laminate. For the SMA hybrid laminates, it is easily seen that the ply mode III laminate shows the highest peak contact force. Compared to the reference laminate, the degree of the load drops is lighter for the hybrid laminates, especially for the ply mode III laminate, which reveals that the damage in the hybrid laminates is less. The comparison of the force-time curves in the rebound phase, indicates that the impactor is rebounded faster in the hybrid laminates, similar to the results of 30 J impacts. The displacement-time curves show that the displacement of the reference laminate is higher than the SMA hybrid laminates. Specifically, the displacement of the ply mode III laminate is the smallest. The energy-time history reveals that the absorbed energy of the ply mode IV and V laminates makes no large difference to the reference laminate. In terms of the rebound energy of the ply mode II and III laminates, both values are larger than the conventional laminate.

**Figure 11:**
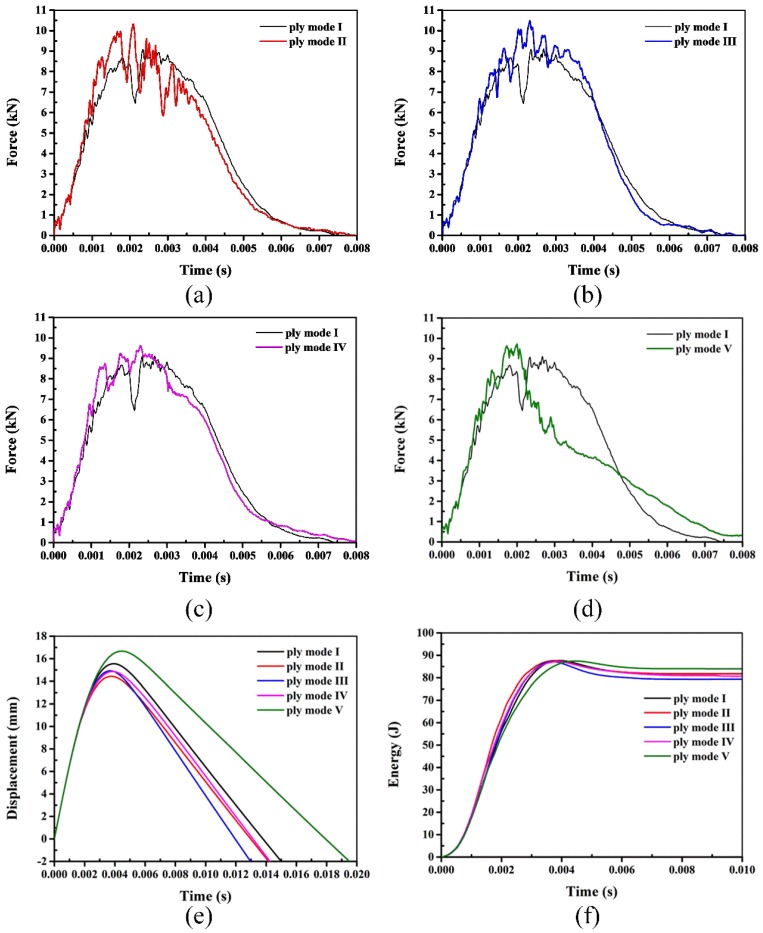
Typical impact behavior of laminates with 90 J (Figures (**a**–**d**) show the force-time curves of mode I with mode II, mode III, mode IV and mode V, respectively. Figure (**e**) and Figure (**f**) represent the displacement-time and energy-time histories of different modes, respectively).

The impact response of the laminates subjected to 90 J is plotted in Figure 11. The results reveal that the laminates are still not penetrated and there are relatively great differences in impact behavior between the laminates reinforced with SMA wires and the reference laminate. For all laminates incorporated with SMA wires, the peak forces are larger than the reference laminate. There is a large load drop in the force-time curve of the reference laminate, which represents the appearance of macroscopic damage during the impact event. Refer to the ply mode II laminate, there are more fluctuations in the force-time curve. For the ply mode III and IV laminates, the fluctuations are less drastic compared to the ply mode II. During the pre-rebound phase, the contact force of ply mode IV sample is generally larger than the reference laminate, and the difference is increased for the ply mode III. Although the peak force of the ply mode V sample is larger than the reference laminate, the contact force decreases largely, even smaller than the reference laminate after the peak force. The displacement-time curves indicate that the displacement of the ply mode V laminate is larger than the reference laminate and the displacement of the ply mode II is the smallest. For the energy-time history, the absorbed energy of the ply mode III laminate is the smallest, the ply mode II, IV and the reference laminates basically have the same absorbed energy, the ply mode V sample has the largest absorbed energy.

## 4. Discussions

### 4.1. Damage Evolution

During an impact event, different damage modes occur and propagate with load increasing. For the laminate without SMA wires, the damage evolution can be described as the following. Firstly, the laminate can be regarded as an elastic plate when the load imposed on the laminate is low. There is almost no damage induced in the laminate. Secondly, micro-cracks occur in the polymer matrix. The fiber/matrix debonding also appears due to the relatively poor strength of the interface between fibers and the polymer matrix. With load increasing, micro-cracks can join together to form main matrix cracks or link fiber/matrix debondings, resulting in matrix crack propagation in the transverse direction. As reported by the work of Yang et al. [31], the results clearly describe the matrix crack propagation process in a microscopic view. For the case of tension, fiber/matrix debondings at different locations are linked by matrix cracks. While in the case of compression, matrix cracks at different locations are linked to form a main crack. With more matrix cracks formed, the accumulated damage in the composite leads to a load drop shown in the contact force-time history. This damage mode can be seen on the ply mode I laminate in Figure 5. As indicated in the research of Wagih et al. [32], the matrix cracks growing at the contact area cause the sudden reduction of the shear stiffness, resulting in a load drop in the force-time curve. Thirdly, when matrix cracks extend to the interface between plies, the delamination is induced. The delamination develops dominated by the orientation of the bottom ply, which has been indicated in [33]. The damage morphology of the ply mode I sample in Figure 6 clearly shows the delamination. Lastly, with extensive propagation of matrix cracks and delamination, fiber breakage occurs when the load imposed is beyond its strength, resulting in a dramatic damage to the laminate. In Figure 7, two main fiber breakage paths almost join together at the center of the ply mode I laminate, which means the structure nearly loses the load bearing ability. For the laminate reinforced with SMA wires, the damage evolution is similar to the reference laminate except two points. One is the SMA/matrix debonding damage mode, which has been illustrated by the SEM images of the SMA hybrid laminate. When the cohesive strength is reached at the interface between SMA wires and the polymer matrix, the SMA/matrix debondings occur and cracks extend along the SMA/matrix interface. Another is the local flexural deformation of the hybrid laminates compared to the relatively brittle damage of the reference laminate. The failure strain of SMA wires used in this work can reach 12.4%, which renders them the large plastic deformation capability. In particular, when SMA wires are incorporated into the lower part of the structure, their superior mechanical performance can be effectively exploited.

### 4.2. Analysis of Impact Responses

The impact resistance of polymer laminates can be evaluated by impact parameters, such as the peak contact force, the maximum displacement and the absorbed energy. Based on the experimental results, the average values of the impact parameters and corresponding increases to the reference laminate over the three impact energy levels (30, 60 and 90 J) are summarized in Table 4. In general, the peak forces of the laminates reinforced with SMA wires are larger than the conventional laminate, while the displacement and absorbed energy of SMA hybrid laminates are smaller than the reference laminate.

During an impact event, the load bearing capability of the polymer composite can be evaluated by the peak force [18]. In the case of 30 J impacts, compared to the reference laminate, the peak force increases by 2.07%, 4.13%, 4.82% and 3.44% for the ply mode II, III, IV and V hybrid laminates, respectively. For 60 J impacts, there are much larger increases in the peak forces of SMA hybrid laminates compared to the case of 30 J impact. Specifically, the ply mode III laminate has the largest improvement of 21.04% relative to the reference laminate, followed by the ply mode II of 17.57%, the ply mode V of 13.61%, and last the ply mode IV of 7.80%. For the samples impacted with 90 J, the ply mode II, III, IV and V laminates show 10.33%, 10.49%, 9.62% and 9.73% increases to the laminate without SMA wires, respectively. The experimental results consistently show that the peak force of SMA hybrid samples is larger than the reference sample. The SMA wires used in this work have a high tensile strength of 1522.7 MPa and a large failure strain of 12.4%. In the composites reinforced with SMA wires, the stress can be transferred from the polymer matrix to the SMA wires. The excellent mechanical performance of SMA wires make a positive influence on the improvement of the tensile strength of the SMA hybrid composites. As reported in our previous study [34], for SMA hybrid basalt fiber-reinforced composite incorporated with SMA wires of 4.62% in volume fraction, the enhancement of tensile strength can reach 10.91%, which enhances the load bearing capability of the SMA hybrid laminates for the fact that the lower part of the structure is subjected to tensile stress when impact loading is imposed on the structure. Moreover, due to the stress induced martensitic phase transformation, SMA wires can keep a nearly high constant stress level over a large strain [17,21], which is also beneficial to the improvement on the load bearing capacity of the SMA hybrid composites. In this work, SMA wires are in austenitic parent phase at room temperature, when the wires are loaded beyond the critical stress 530.8 MPa, the austenite starts to transform to martensite, which reverts back to austenite once the load is removed. During the martensitic phase transformation process, SMA wires can keep a nearly high constant stress level of 530.8 MPa over a large strain of about 5.7%, providing them with the ability to resist a large amount of load. Therefore, the load bearing capability of SMA hybrid composites is improved due to the reinforcement of SMA wires. Compared to conventional composite laminates, larger peak forces are also observed for SMA hybrid graphite laminates [18] and glass fiber-reinforced composites [35] subjected to low-velocity impact. The higher load bearing capability indicates an advantage in the ability of the SMA hybrid laminates to dissipate impact energy over the conventional laminate. The higher load resistance capability of SMA wires prevents the initiation and propagation of damage during an impact event, as a consequence, the SMA hybrid laminates have higher peak forces and impact tolerance.

The maximum displacement can be used as another indication for the impact resistance of the laminate [30]. The laminate with higher stiffness will produce smaller displacement when subjected to impact load. For 30 J impacts, the displacements of laminates with SMA wires are smaller than the laminate without SMA wires. The ply mode IV and V laminates show better resistance to deformation and decrease by about 6.3% in maximum displacement to the reference laminate. For the laminates impacted with 60 J, the ply mode III hybrid laminate makes the most positive effect on the structure deformation and has a significant decrease of 8.6% compared to the conventional laminate. At 90 J the maximum displacement of the ply mode II laminate has a reduction of 7.2% to the base laminate, while the maximum displacement of ply mode V is larger than the laminate without SMA wires. In general, the maximum displacement of SMA hybrid laminates is smaller than the reference laminate, which indicates that the hybrid laminates are stiffer and impact resistance is enhanced due to the incorporation of SMA wires. When composite laminates are imposed with impact loading, the flexural strength makes significant effect on the structural deformation. Due to the higher load resistance capability of SMA wires embedded into the composites, the flexural strength of SMA hybrid laminates is enhanced and hence the structural deformation is decreased. As indicated in our previous study [34], for SMA hybrid basalt fiber-reinforced composite embedded with SMA wires at the lower part of the structure, the improvement of flexural strength can reach 40.31% with only 4.19% volume fraction SMA wires. Compared to conventional glass fiber-reinforced composites, smaller maximum displacement is also observed for SMA hybrid composites subjected to low velocity impact [30]. In particular, the SMA wires along the bottom ply produce the smallest displacement in their work. The displacement-time curves also indicate that the impactor returns the contact position faster for the SMA hybrid samples, especially, the time of the ply mode III laminate is 9.14 ms compared to 11.64 ms of the reference laminate for 60 J impacts. This phenomenon reveals less damage induced in the SMA hybrid samples relative to the reference sample, which means that the impact resistance of SMA hybrid composites is enhanced. In addition, the recovery stress produced by SMA wires makes positive effect on the recover to the initial location of the structure. In the work of Rim et al. [15], SMA hybrid laminates also deflect less than conventional composite plates and more recovered deflection of the hybrid laminates is observed.

When the composite laminate encounters impact loading, the impact energy is absorbed by the laminate through elastic deformation or plastic deformation or various failure processes [5]. The energy dissipated by the elastic deformation of the laminate is released by rebounding the impactor. Basalt fiber-reinforced composites however experience very little or no plastic deformation due to the low strain to failure of the fiber and brittle nature of the epoxy matrix. Hence, the impact energy is mainly dissipated through different damage modes. As shown in Table 3, in general the absorbed energy of SMA hybrid samples is smaller than the conventional sample, which indicates that the energy causing damage is more in the reference laminate. In this work, the high tensile strength and failure strain of SMA wires render them the ability to deform largely, and during the stress induced martensitic phase transformation process, the recoverable strain of SMA wires is up to 7.9%, resulting in a large amount of impact energy absorbed by the SMA wires. As pointed out in researches [35,36], the energy leading to damage is reduced in the SMA hybrid laminates due to the energy absorbing capability of the SMA wires. Moreover, the SMA/matrix debonding can dissipate some impact energy, and after the SMA/matrix debonding, the friction between SMA wires and the surrounding polymer can also make a positive effect on the energy dissipation. All these energy dissipation mechanisms benefit the impact resistance of the laminates incorporated with SMA wires, as a consequence, there is less damage and the impact resistance is enhanced for SMA hybrid samples.

Based on the above discussion, a combination of the peak force, the maximum displacement and the absorbed energy, reveals that the best impact resistance of SMA hybrid composite can be acquired using the ply mode III. In particular, the improvement is the largest when the 60 J impact energy is imposed. Hence, to make the most of SMA hybrid basalt fiber-reinforced polymer composites embedded with two perpendicular layers of superelastic SMA wires, the ply mode III, namely with one layer of SMA wires placed between the front two plies and another layer placed between the bottom two plies, can be a useful selection for design.

## 5. Conclusions

The low velocity impact behavior of the new basalt fiber-reinforced polymer composites incorporated with two perpendicular layers of superelastic SMA wires is experimentally investigated. Tests at three impact energy levels (30, 60 and 90 J) are carried out and four different SMA wires embedded modes are investigated.

The following conclusions can be drawn:

(1) Compared to the conventional composite laminate, the load bearing capability of the SMA hybrid laminates is enhanced due to the higher load resistance capability of SMA wires. The higher load bearing capability indicates that more impact energy can be dissipated, leading to the improvement of the impact tolerance of the SMA hybrid composites.
(2) Compared to the reference laminate, the maximum displacement of SMA hybrid laminates is smaller, and it is faster for the impactor to return the contact position during the hybrid laminate impact event.
(3) A large amount of impact energy is dissipated by SMA wires mainly due to their stress induced martensitic transformation energy absorbing mechanism, leaving less energy to cause damage in the SMA hybrid laminates. Therefore, the impact resistance of hybrid laminates is enhanced.
(4) The comparison of the impact response of the hybrid laminates with SMA wires embedded at different positions, shows that the most effective impact resistance can be obtained in the hybrid ply mode III laminate with one layer of SMA wires placed between the front two plies and another layer placed between the bottom two plies. In particular, the ply mode III laminate shows an improvement up to 21.04% in the peak force, a reduction of 8.6% in the maximum displacement and a decrease of 7.35% in the absorbed energy, compared to the reference composite laminate for 60 J impacts.

## Figures and Tables

**Table 1:** The mechanical properties of the materials used in the polymer composites.

Materials	Modulus (GPa)	Tensile Strength (MPa)	Failure Strain (%)
Vinyl ester	3.2	86	4.9
Basalt fiber	95	3080	3.1

**Table 2:** The main mechanical characteristics of SMA wires.

Material	Modulus (GPa)	Tensile Strength (MPa)	Failure Strain (%)	Upper Plateau Stress (MPa)	Lower Plateau Stress (MPa)	Recoverable Strain (%)
NiTi	60.6	1522.7	12.4	530.8	170.8	7.9

**Table 3:** The exact locations of the embedded SMA fibers in composites.

Category	Ply Mode	Stacking Sequence
Without SMA	Ply mode І	0°/0°/90°/90°/0°/0°/90°/90°/0°/0°/90°/90°
With SMA	Ply mode ІІ	0°/SMA/0°/90°/SMA/90°/0°/0°/90°/90°/0°/0°/90°/90°
	Ply mode ІІІ	0°/SMA/0°/90°/90°/0°/0°/90°/90°/0°/0°/90°/SMA/90°
	Ply mode ІV	0°/0°/90°/SMA/90°/0°/0°/90°/90°/0°/SMA/0°/90°/90°
	Ply mode V	0°/0°/90°/90°/0°/0°/90°/90°/0°/SMA/0°/90°/SMA/90°

**Table 4:** Average values of the impact response and the increase compared to the reference laminate.

Ply Mode	Impact Energy (J)	Peak Force (kN)/Increase		Displacement (mm)/Increase		Energy (J)/Increase	
I	30	7.26 ± 0.05	-	8.81 ± 0.04	-	20.15 ± 0.05	-
II		7.41 ± 0.1	2.07%	8.44 ± 0.06	−4.20%	19.19 ± 0.09	−4.75%
III		7.56 ± 0.09	4.13%	8.49 ± 0.08	−3.63%	19.23 ± 0.1	−4.57%
IV		7.61 ± 0.06	4.82%	8.25 ± 0.05	−6.36%	18.72 ± 0.04	−7.09%
V		7.51 ± 0.07	3.44%	8.26 ± 0.07	−6.24%	19.07 ± 0.08	−5.36%
I	60	8.08 ± 0.1	-	12.32 ± 0.07	-	52.68 ± 0.06	-
II		9.50 ± 0.2	17.57%	11.64 ± 0.09	−5.52%	49.93 ± 0.08	−5.22%
III		9.78 ± 0.2	21.04%	11.26 ± 0.1	−8.60%	48.81 ± 0.1	−7.35%
IV		8.71 ± 0.2	7.80%	12.01 ± 0.1	−2.52%	52.36 ± 0.09	−0.61%
V		9.18 ± 0.09	13.61%	12.07 ± 0.06	−2.03%	51.75 ± 0.05	−1.77%
I	90	9.11 ± 0.1	-	15.55 ± 0.1	-	82.17 ± 0.07	-
II		10.33 ± 0.2	13.40%	14.43 ± 0.2	−7.20%	81.63 ± 0.2	−0.66%
III		10.49 ± 0.2	15.15%	14.78 ± 0.2	−4.95%	79.21 ± 0.1	−3.60%
IV		9.62 ± 0.08	5.60%	14.86 ± 0.1	−4.44%	80.62 ± 0.06	−1.89%
V		9.73 ± 0.3	6.81%	16.67 ± 0.3	7.20%	83.65 ± 0.2	1.80%

## References

[1] Sarasini F., Tirillò J., Valente M., Valente T., Cioffi S., Iannace S., Sorrentino L. (2013). Effect of basalt fibre hybridization on the impact behaviour under low impact velocity of glass/basalt woven fabric/epoxy resin composites. Compos. Part A Appl. Sci. Manuf..

[2] Lopes C.S., Seresta O., Coquet Y., Gürdal Z., Camanho P.P., Thuis B. (2009). Low-velocity impact damage on dispersed stacking sequence laminates. Part I Exp. Compos. Sci. Technol..

[3] Riccio A., De Luca A., Di Felice G., Caputo F. (2014). Modelling the simulation of impact induced damage onset and evolution in composites. Compos. Part B Eng..

[4] Aymerich F., Dore F., Priolo P. (2009). Simulation of multiple delaminations in impacted cross-ply laminates using a finite element model based on cohesive interface elements. Compos. Sci. Technol..

[5] Fragassa C., Pavlovic A., Santulli C. (2018). Mechanical and impact characterisation of flax and basalt fibre vinylester composites and their hybrids. Compos. Part B Eng..

[6] Verrey J., Winkler Y., Michaud V., Månson J.A. (2005). Interlaminar fracture toughness improvement in composites with hyperbranched polymer modified resin. Compos. Sci. Technol..

[7] Chandrasekaran V.C.S., Advani S.G., Santare M.H. (2011). Influence of resin properties on interlaminar shear strength of glass/epoxy/MWNT hybrid composites. Compos. Part A Appl. Sci. Manuf..

[8] van der Heijden S., Daelemans L., Meireman T., De Baere I., Rahier H., Van Paepegem W., De Clerck K. (2016). Interlaminar toughening of resin transfer molded laminates by electrospun polycaprolactone structures: Effect of the interleave morphology. Compos. Sci. Technol..

[9] Van Velthem P., Ballout W., Daoust D., Sclavons M., Cordenier F., Henry E., Dumont D., Destoop V., Pardoen T., Bailly C. (2015). Influence of thermoplastic diffusion on morphology gradient and on delamination toughness of RTM-manufactured composites. Compos. Part A Appl. Sci. Manuf..

[10] Arnold M., Henne M., Bender K., Drechsler K. (2015). The influence of various kinds of PA12 interlayer on the interlaminar toughness of carbon fiber-reinforced epoxy composites. Polym. Compos..

[11] Yudhanto A., Watanabe N., Iwahori Y., Hoshi H. (2013). Effect of stitch density on tensile properties and damage mechanisms of stitched carbon/epoxy composites. Compos. Part B Eng..

[12] Blacklock M., Joosten M.W., Pingkarawat K., Mouritz A.P. (2016). Prediction of mode I delamination resistance of z-pinned laminates using the embedded finite element technique. Compos. Part A Appl. Sci. Manuf..

[13] Petrucci R., Santulli C., Puglia D., Sarasini F., Torre L., Kenny J.M. (2013). Mechanical characterisation of hybrid composite laminates based on basalt fibres in combination with flax, hemp and glass fibres manufactured by vacuum infusion. Mater. Des..

[14] Nisini E., Santulli C., Liverani A. (2017). Mechanical and impact characterization of hybrid composite laminates with carbon, basalt and flax fibres. Compos. Part B Eng..

[15] Rim M.S., Kim E.H., Lee I., Choi I.H., Ahn S.M., Koo K.N., Bae J.S., Roh J.H. (2011). Low-velocity impact characteristics of composite plates with shape memory alloy wires. J. Theor. Appl. Mech. Pol..

[16] Pappadà S., Rametta R., Toia L., Coda A., Fumagalli L., Maffezzoli A. (2009). Embedding of superelastic SMA wires into composite structures: Evaluation of impact properties. J. Mater. Eng. Perform..

[17] Raghavan J., Bartkiewicz T., Boyko S., Kupriyanov M., Rajapakse N., Yu B. (2010). Damping, tensile, and impact properties of superelastic shape memory alloy (SMA) fiber-reinforced polymer composites. Compos. Part B Eng..

[18] Paine J.S.N., Rogers C.A. (1994). The response of SMA hybrid composite materials to low velocity impact. J. Intell. Mater. Syst. Struct..

[19] Aurrekoetxea J., Zurbitu J., de Mendibil I.O., Agirregomezkorta A., Sánchez-Soto M., Sarrionandia M. (2011). Effect of superelastic shape memory alloy wires on the impact behavior of carbon fiber reinforced in situ polymerized poly (butylene terephthalate) composites. Mater. Lett..

[20] Lau K.T., Ling H.Y., Zhou L.M. (2004). Low velocity impact on shape memory alloy stitched composite plates. Smart Mater. Struct..

[21] Kang K.W., Kim J.K. (2009). Effect of shape memory alloy on impact damage behavior and residual properties of glass/epoxy laminates under low temperature. Compos. Struct..

[22] Khalili S.M.R., Shokuhfar A., Malekzadeh K., Ghasemi F.A. (2007). Low velocity impact response of active thin-walled hybrid composite structures embedded with SMA wires. Thin Wall. Struct..

[23] Yang B., Wang Z.Q., Zhou L.M., Zhang J.F., Tong L.Y., Liang W.Y. (2015). Study on the low-velocity impact response and CAI behavior of foam-filled sandwich panels with hybrid facesheet. Compos. Struct..

[24] Lei H.S., Wang Z.Q., Tong L.Y., Zhou B., Fu J. (2013). Experimental and numerical investigation on the macroscopic mechanical behavior of shape memory alloy hybrid composite with weak interface. Compos. Struct..

[25] Wang Z., Liu Y., Lv H., Yang B. (2017). Enhancement of interface performance between shape memory alloy fiber and polymer matrix using silane coupling agent KH550 and Al_2_O_3_ nanoparticles. Polym. Compos..

[26] Neuking K., Abu-Zarifa A., Eggeler G. (2009). Surface engineering of shape memory alloy/polymercomposites: Improvement of the adhesion between polymers and pseudoelastic shape memory alloys. Mater. Sci. Eng. A.

[27] Pequegnata A., Michael A., Wang J., Lian K., Zhou Y., Khan M.I. (2015). Surface characterizations of laser modified biomedical grade NiTi shape memory alloys. Mater. Sci. Eng. C.

[28] Rossi S., Deflorian F., Pegoretti A., D’Orazio D., Gialanella S. (2008). Chemical and mechanical treatments to improve the surface properties of shape memory NiTi wires. Surf. Coat. Technol..

[29] Wong M.H., Cheng F.T., Pang G.K.H., Man H.C. (2007). Characterization of oxide film formed on NiTi by laser oxidation. Mater. Sci. Eng. A.

[30] Tsoi K.A., Stalmans R., Schrooten J., Wevers M., Mai Y.W. (2003). Impact damage behaviour of shape memory alloy composites. Mater. Sci. Eng. A.

[31] Yang L., Yan Y., Liu Y., Ran Z. (2012). Microscopic failure mechanisms of fiber-reinforced polymer composites under transverse tension and compression. Compos. Sci. Technol..

[32] Wagih A., Maimí P., Blanco N., Costa J. (2016). A quasi-static indentation test to elucidate the sequence of damage events in low velocity impacts on composite laminates. Compos. Part. A Appl. Sci. Manuf..

[33] Lopes C.S., Camanho P.P., Gürdal Z., Maimí P., González E.V. (2009). Low-velocity impact damage on dispersed stacking sequence laminates. Part II: Numerical simulations. Compos. Sci. Technol..

[34] Liu Y., Wang Z., Li H., Sun M., Wang F., Chen B. (2018). Influence of Embedding SMA Fibres and SMA Fibre Surface Modification on the Mechanical Performance of BFRP Composite Laminates. Materials.

[35] Sun M., Wang Z., Yang B., Sun X. (2017). Experimental investigation of GF/epoxy laminates with different SMAs positions subjected to low-velocity impact. Compos. Struct..

[36] Sofocleous K., Ogin S.L., Tsakiropoulos P., Draconakis V., Doumanidis C. (2014). Controlled impact testing of woven fabric composites with and without reinforcing shape-memory alloy wires. J. Compos. Mater..

